# Supplementary material for: Serological identification of SARS-CoV-2 infections among children visiting a hospital during the initial Seattle outbreak
Source: Nat Commun. 2020 Sep 1;11:4378. doi: 10.1038/s41467-020-18178-1 (PMC7463158; doi:10.1038/s41467-020-18178-1)
Supplement: Supplementary file 3 — Descriptions of Additional Supplementary Files [file 41467_2020_18178_MOESM3_ESM.pdf]

Description of Additional Supplementary Files

Supplementary Data 1

Description: De-identified clinical data, raw OD450 values from RBD IgG ELISA screen, AUCs from follow up ELISAs, and Abbott index values for each sample.

Supplementary Data 2

Description: Raw OD450 values across all titrations from RBD and spike follow-up IgG ELISAs.

Supplementary Data 3

Description: Fraction infectivity values across all titrations from spike-pseudotyped lentivirus neutralization assays.

Supplementary Data 4

Description: Summary IC50 values, RBD and Spike IgG ELISA AUC values, and Abbot index values for all samples run in spike-pseudotyped lentivirus neutralization assays.
