# Supplementary material for: Serological identification of SARS-CoV-2 infections among children visiting a hospital during the initial Seattle outbreak
Source: Nat Commun. 2020 Sep 1;11:4378. doi: 10.1038/s41467-020-18178-1 (PMC7463158; doi:10.1038/s41467-020-18178-1)
Supplement: Supplementary file 8 — Reporting Summary [file 41467_2020_18178_MOESM8_ESM.pdf]

nature research

Corresponding author(s): Jesse Bloom

Last updated by author(s): 07/31/2020

Reporting Summary

Nature Research wishes to improve the reproducibility of the work that we publish. This form provides structure for consistency and transparency in reporting. For further information on Nature Research policies, see our Editorial Policies and the Editorial Policy Checklist.

Statistics

For all statistical analyses, confirm that the following items are present in the figure legend, table legend, main text, or Methods section.

n/a	Confirmed	
☐	☒	The exact sample size (n) for each experimental group/condition, given as a discrete number and unit of measurement
☒	☐	A statement on whether measurements were taken from distinct samples or whether the same sample was measured repeatedly
☐	☒	The statistical test(s) used AND whether they are one- or two-sided<br>*Only common tests should be described solely by name; describe more complex techniques in the Methods section.*
☒	☐	A description of all covariates tested
☒	☐	A description of any assumptions or corrections, such as tests of normality and adjustment for multiple comparisons
☐	☒	A full description of the statistical parameters including central tendency (e.g. means) or other basic estimates (e.g. regression coefficient) AND variation (e.g. standard deviation) or associated estimates of uncertainty (e.g. confidence intervals)
☒	☐	For null hypothesis testing, the test statistic (e.g. F, t, r) with confidence intervals, effect sizes, degrees of freedom and P value noted<br>*Give P values as exact values whenever suitable.*
☒	☐	For Bayesian analysis, information on the choice of priors and Markov chain Monte Carlo settings
☒	☐	For hierarchical and complex designs, identification of the appropriate level for tests and full reporting of outcomes
☐	☒	Estimates of effect sizes (e.g. Cohen's d, Pearson's r), indicating how they were calculated

Our web collection on statistics for biologists contains articles on many of the points above.

Software and code

Policy information about availability of computer code

Data collection	No software was used to collect data.
Data analysis	Simple, custom Python scripts were used to analyze the data, but these are not publicly available because it includes identifiable information (dates linked with samples). All de-identified raw data is available as supplementary items; the overall results of this study can be easily recreated from these, with the only exception being that de-identified data only include 2-week timing intervals for samples. Other software used during analysis included our neutcurve package (https://jbloomlab.github.io/neutcurve/, version 0.3.1).

For manuscripts utilizing custom algorithms or software that are central to the research but not yet described in published literature, software must be made available to editors and reviewers. We strongly encourage code deposition in a community repository (e.g. GitHub). See the Nature Research guidelines for submitting code & software for further information.

Data

Policy information about availability of data

All manuscripts must include a data availability statement. This statement should provide the following information, where applicable:

- Accession codes, unique identifiers, or web links for publicly available datasets
- A list of figures that have associated raw data
- A description of any restrictions on data availability

All raw serological data and de-identified demographic and clinical data are included as supplemental items (corresponding to Supplementary Data 1-4). Complete clinical data and exact dates is restricted for privacy concerns as described in the Data Availability section.

Field-specific reporting

Please select the one below that is the best fit for your research. If you are not sure, read the appropriate sections before making your selection.

☒ Life sciences ☐ Behavioural & social sciences ☐ Ecological, evolutionary & environmental sciences

For a reference copy of the document with all sections, see nature.com/documents/nr-reporting-summary-flat.pdf

Life sciences study design

All studies must disclose on these points even when the disclosure is negative.

Sample size	No sample-size calculation was performed. Sample size was determined solely by the number of residual sera samples that were collected at Seattle Children's Hospital and how much volume remained in those samples after their medical use.
Data exclusions	Residual sera samples from patients who were recruited to come to Seattle Children's to enroll in a COVID-19 study were excluded from the study population, as described in the methods. Theses exclusions were pre-established, and our study population was only the residual sera samples from non-recuited individuals at Seattle Children's, as described in the manuscript.
Replication	Reproducibility of experimental results was was assessed during assay development. Reproducibility of experimental findings of this study was only addressed on an ad-hoc basis for a limited number of samples; all results (e.g. serostatus) were qualitatively consistent. Additionally, three separate ELISA assays were well correlated, and the neutralization assay further validated our serostatus calls. Neutralization assays were run in technical duplicate. Abbot CMIA were performed a single time according to clinical lab practice.
Randomization	Samples were not randomized, as investigators were already blinded to RT-PCR testing status; further randomization would not have further blinded the researchers.
Blinding	Investigators were blinded to RT-PCR testing status of nearly all samples until after running serological tests. However, in a small number of cases (n=2 patients in the study), investigators knew the patients had tested positive by RT-PCR because of the sample labeling scheme. Neutralization assays and Abbot CMIA were performed blinded to the ELISA and RT-PCR data.

Reporting for specific materials, systems and methods

We require information from authors about some types of materials, experimental systems and methods used in many studies. Here, indicate whether each material, system or method listed is relevant to your study. If you are not sure if a list item applies to your research, read the appropriate section before selecting a response.

Materials & experimental systems

n/a	Involved in the study
☐	☒ Antibodies
☐	☒ Eukaryotic cell lines
☒	☐ Palaeontology and archaeology
☒	☐ Animals and other organisms
☐	☒ Human research participants
☒	☐ Clinical data
☒	☐ Dual use research of concern

Methods

n/a	Involved in the study
☒	☐ ChIP-seq
☒	☐ Flow cytometry
☒	☐ MRI-based neuroimaging

Antibodies

Antibodies used	CR3022, produced in house. Goat anti-human IgG-Fc horseradish peroxidase (HRP)-conjugated antibody (Bethyl Labs, A80-104P).
Validation	CR3022 was confirmed to bind RBD and spike by ELISA, as shown in Figure 1A. Sera samples or this positive control was the primary antibody. The secondary antibody is validated using CR3022, known positive adult sera, and pre-2020 pooled negtaive control sera, as shown in Figure 1.

Eukaryotic cell lines

Policy information about cell lines

Cell line source(s)	HEK-293T-hACE2. Generated by our lab but available from and externally validated by BEI resources (NR-52511). Expi293F were from Thermo Fisher (A14527).
Authentication	Confirmed hACE2 expression by Flow staining (published in DOI:10.3390/v12050513)

Mycoplasma contamination	Cell line was not tested mycoplasma testing in house; testingby BEI pending.
Commonly misidentified lines (See ICLAC register)	No commonly misidentified cell lines were used in the study.

Human research participants

Policy information about studies involving human research participants

Population characteristics	Samples were de-identified residual sera collected at Seattle Children's Hospital from March 3 to April 24, 2020. Demographics are described in the manuscript in Table 1 and Supplementary Data. The adult control samples were ed-identified.
Recruitment	Patients were not recruited to the study population, as designed and described in the manuscript. Adequate volume of sera remaining after other lab tests were conducted was the only sample-selection criterion, which inherently reduces the relative number of samples from infants who have smaller blood-draw volumes. However, this only reduces our power to detect seropositive cases among this age group, it does not bias the results.
Ethics oversight	The sample collection and this study were approved by the Institutional Review Boards of Seattle Children's Hospital and the University of Washington. This study was granted a waiver of consent since it used residual clinical samples and existing clinical data, and this is stated in the manuscript.

Note that full information on the approval of the study protocol must also be provided in the manuscript.
